# Successful surgical internal drainage of postoperative pancreatic pseudocyst through pancreaticojejunostomy with distal pancreatectomy: a case report

**DOI:** 10.1186/s40792-015-0057-x

**Published:** 2015-06-26

**Authors:** Kazuya Sakata, Daisuke Hashimoto, Katsunobu Taki, Osamu Nakahara, Masaki Ohmuraya, Akira Chikamoto, Toru Beppu, Hideo Baba

**Affiliations:** Department of Gastroenterological Surgery, Kumamoto University Graduate School of Medical Sciences, 1-1-1 Honjo, Kumamoto, 860-8556 Japan; Department of Surgery, Taragi Municipal Hospital, 4210 Taragi, Kumamoto, 868-0598 Japan; Institute of Resource Development and Analysis, Kumamoto University, 2-2-1 Honjo, Kumamoto, 860-0811 Japan

**Keywords:** Pancreatic pseudocysts, Distal pancreatectomy, Pancreaticojejunostomy

## Abstract

Pancreatic pseudocyst is usually treated by percutaneous external drainage, endoscopic internal or external drainage, or surgical internal drainage such as cystogastrostomy. Surgical external drainage is an option if these procedures fail. We describe a case of a 70-year-old man with a pancreatic body pseudocyst that developed postoperatively. It was improved by endoscopic external drainage, and the stent was changed to an internal stent. However, surgery was required as the pseudocyst grew again. A direct approach to the pseudocyst was not possible because of severe adhesion. A distal pancreatectomy with pancreaticojejunostomy was performed, and an external pancreatic stent tube was inserted from the cut end into the duodenum to drain the pseudocyst. One month later, the pseudocyst disappeared, and the stent was removed.

## Background

Pancreatic pseudocyst (PPC) is associated with acute pancreatitis and chronic pancreatitis, and develops as a postoperative complication [1]. PPC is a localized collection of amylase-rich fluid located within or adjacent to the pancreas and is devoid of an epithelial wall [2]. Treatment is only required for persisting PPC symptoms such as abdominal pain, infection, or compression of the gastrointestinal tract, pancreatic duct, or the common bile duct [3]. Although it is usually treated by percutaneous or endoscopic drainage [4], surgery is necessary in some cases, which is associated with a relatively high percentage of complications and even death [5]. We herein describe successful surgical external drainage of postoperative PPC through pancreaticojejunostomy with distal pancreatectomy (DP).

## Case presentation

**Fig. 1:**
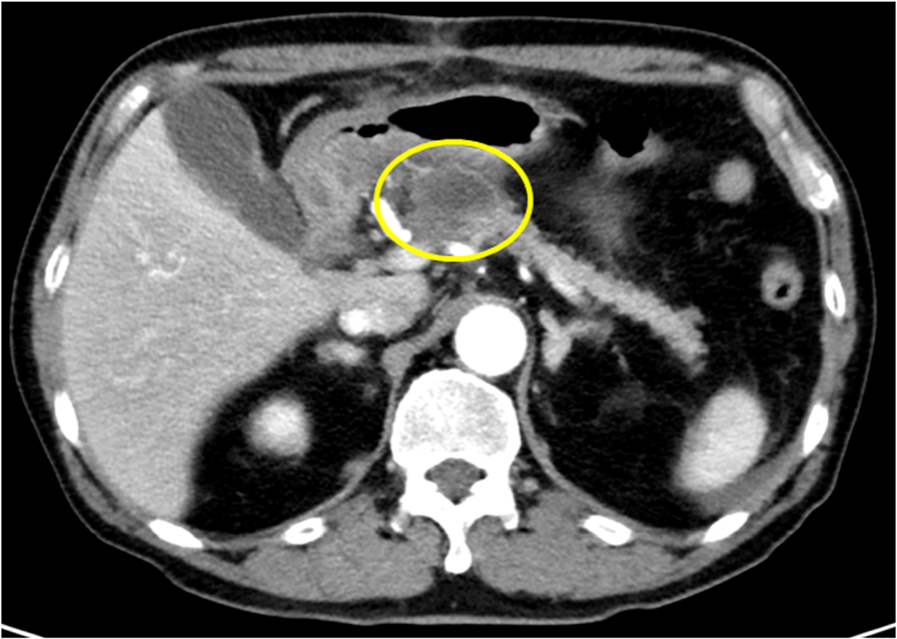
Pancreatic pseudocyst. Acute pancreatitis repeatedly occurred 1 month after the first operation, and a pancreatic pseudocyst developed at the same site. Enhanced computed tomography (CT) showed a cyst with a diameter of 29 mm in the body of the pancreas

**Fig. 2:**
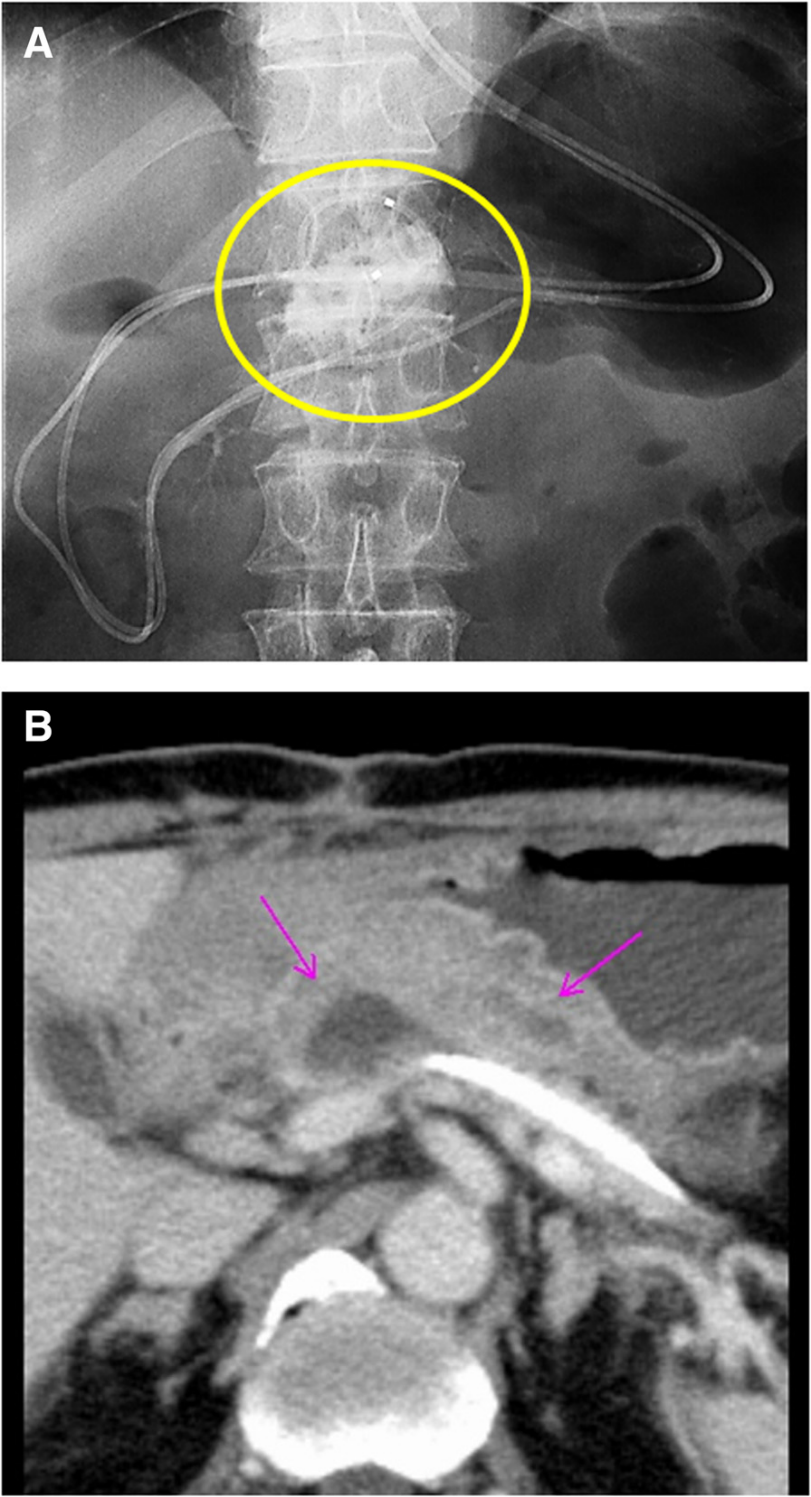
Endoscopic approach for pancreatic pseudocyst. **a** Two drainage tubes were inserted into the pancreatic pseudocyst and main pancreatic duct through the ampulla of Vater. **b** Enhanced CT showed that acute pancreatitis and pancreatic pseudocyst had recurred 1 month after endoscopic drainage

A 70-year-old man underwent partial resection of the mid pancreas without reconstruction for a pancreatic cystic tumor. Postoperative pathological examination showed a lymphoepithelial cyst. About 1 month after the operation, a PPC developed as a consequence of grade B postoperative pancreatic fistula (POPF) and acute pancreatitis (Fig. 1). Internal drainage of the PPC using endoscopic ultrasonography (EUS) should have been considered as one of the procedures. However, there was no doctor who was skilled in the procedure at our hospital, and we wanted to observe the PPC over time. Therefore, transpapillary drainage was judged to be the first choice of treatment. Endoscopic drainage was subsequently performed, and two endoscopic nasopancreatic drainage (ENPD) tubes were placed into the PPC and main pancreatic duct (Fig. 2a). There was a stricture of the main pancreatic duct near the PPC, and we judged that long stent insertion was necessary and considered that endoscopic retrograde pancreatic drainage (ERPD) was more appropriate than ENPD from the view of QOL. After improvement of abdominal pain and PPC was observed, the drainage tubes were exchanged with an ERPD tube.

**Fig. 3:**
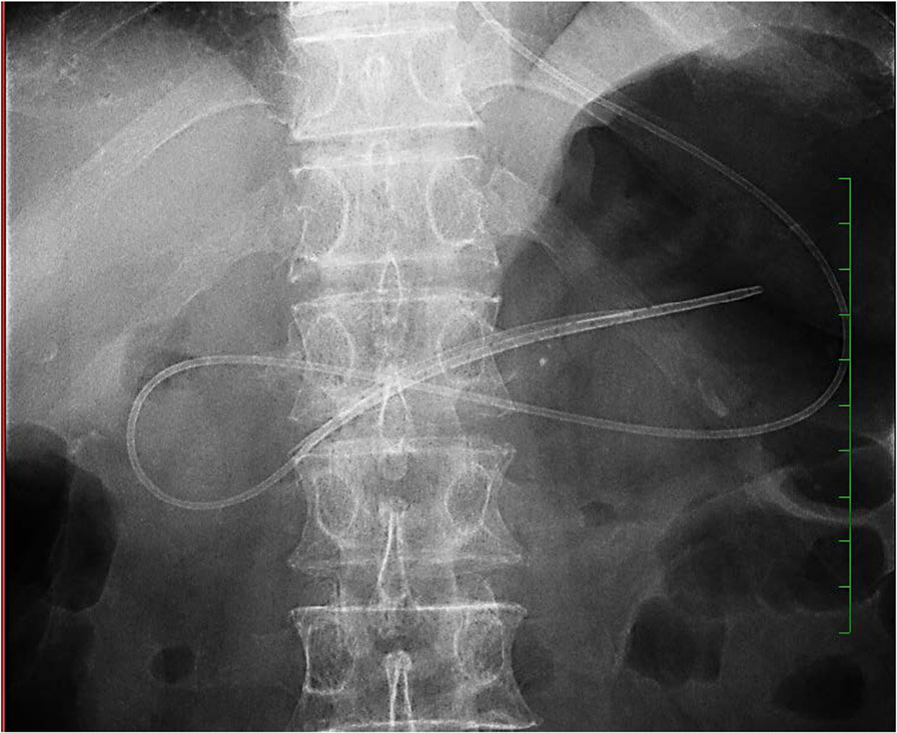
Migration of the ERPD tube into the main pancreatic duct. Endoscopic nasopancreatic drainage (ENPD) tubes were placed again into the main pancreatic duct side by side with the migrated ERPD tube

**Fig. 4:**
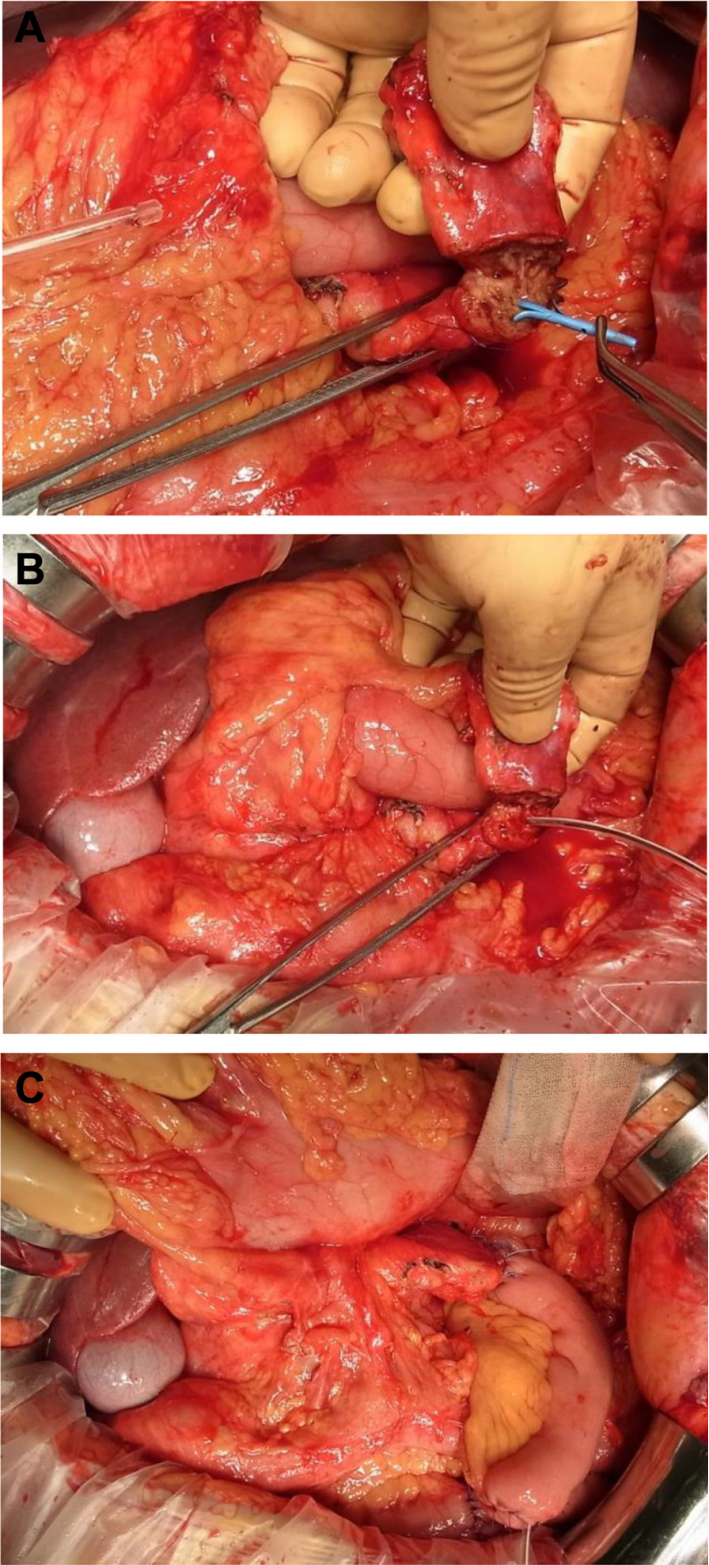
Distal pancreatectomy and pancreatojejunostomy (R-Y reconstruction). **a** The position of the endoscopic retrograde pancreatic drainage tube was checked with US, and the pancreatic body and tail were resected. **b** The pancreatic drainage tube was inserted from the tail to the duodenum. **c** Pancreatojejunostomy was performed to drain the pancreatic body and tail followed by R-Y reconstruction

**Fig. 5:**
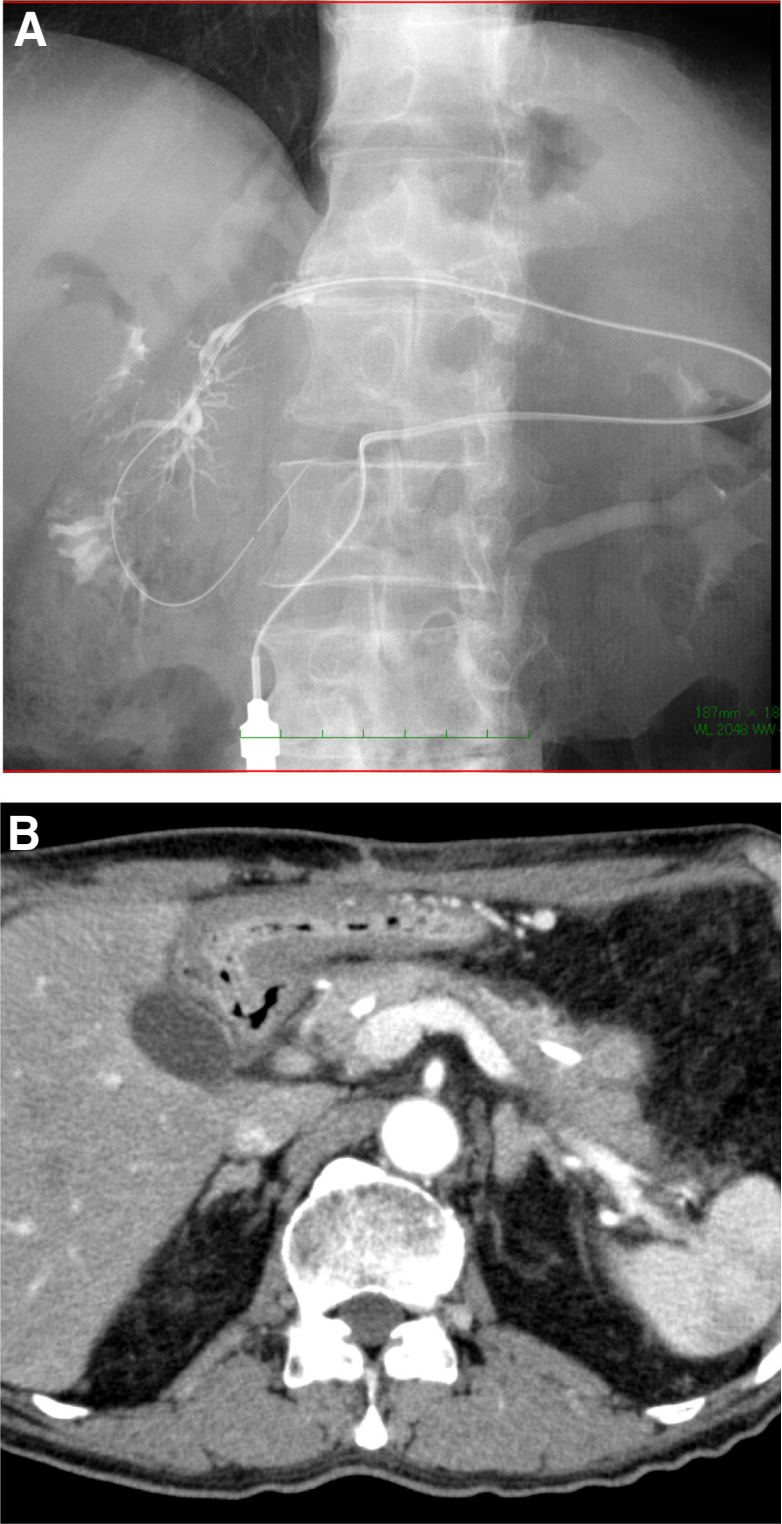
Disappearance of pancreatic pseudocyst. **a** The pancreatic pseudocyst was seen on pancreatography through the pancreatic drainage tube. **b** Enhanced CT showed that the pancreatic pseudocyst had disappeared 4 weeks after the second operation

One month later, the PPC worsened again (Fig. 2b). Unfortunately, the ERPD tube had migrated into the main pancreatic duct and could not be removed endoscopically. ENPD tubes were placed again into the main pancreatic duct side by side with the migrated ERPD tube (Fig. 3). In addition, bleeding from the main pancreatic duct caused by tube contact was observed. If the condition was absent, internal drainage of PPC using endoscopic ultrasonography (EUS) should be considered because additional pancreaticojejunostomy may cause another postoperative pancreatic fistula. The patient underwent a reoperation to remove the ERPD tube and to drain the PPC. Because severe adhesions were present between the PPC and stomach, the PPC could not be approached directly. The pancreatic tail was then mobilized away from the spleen. The position of the endoscopic retrograde pancreatic drainage tube was checked with ultrasonography (US) during the operation, and the pancreatic body and tail were resected on the position (Fig. 4a). The migrated ERPD tube was removed smoothly from the cut end (Fig. 4a). There was a stricture of the main pancreatic duct near the PPC, and the drainage of the pancreatic tail was not effective. Insertion of an external pancreatic drainage stent tube from the cut end into the duodenum through the ampulla of Vater was needed to drain the PPC because we considered the drainage of the pancreatic body to be insufficient (Fig. 4b). A pancreaticojejunostomy was then made between the pancreatic cut end and the jejunum with Roux-Y reconstruction (Fig. 4c). The patient did not have any postoperative complications. One month after discharge, the PPC had disappeared completely (Fig. 5a, b), and the external drainage stent was removed.

PPCs may develop in 10–20 % and 20–40 % of patients with acute and chronic pancreatitis, respectively [6, 7]. Currently, endoscopic drainage is recommended as a first-line treatment for accessible PPCs because the outcomes are excellent in terms of costs, duration of hospital stay, and quality of life, as was demonstrated in a recent prospective randomized study [8]. However, in the present case, surgery was required because endoscopic drainage had failed and hemorrhage occurred.

A variety of surgical techniques exist for PPC [9, 10]. Internal drainage via cystojejunostomy has been the treatment of choice [11]. However, this type of anastomosis was unsuitable in the present case because severe adhesions were seen around the PPC. Adhesiolysis is associated with a high risk of bowel injury [12]. Instead of cystojejunostomy, an external pancreatic drainage tube was inserted from the cut pancreatic tail into the duodenum to treat the PPC. To our knowledge, there are no similar published cases in the English literature.

In our case, the pancreatic cut end was not closed directly but was anastomosed to the jejunum. Klein et al. compared pancreatoenteral anastomosis with direct closure of the pancreatic remnant for POPF after DP and reported that pancreatoenteral anastomosis may be considered a safe alternative for direct closure in certain cases [13]. Pancreatoenteral anastomosis might have contributed to bilateral drainage of pancreatic juice to the head and tail sides in the present case, even if the main pancreatic duct was stenosed.

## Conclusions

Appropriate drainage was important in managing PPC, and external drainage through pancreaticojejunostomy with DP is an effective procedure for PPC if endoscopic treatment is unsuccessful.

## Consent

Written informed consent was obtained from the patient for publication of this case report and any accompanying images. A copy of the written consent is available for review by the Editor-in-Chief of this journal.
